# Regulatory Measures' Effect on Gambling Participation: Experiences From Norway

**DOI:** 10.3389/fpsyt.2021.672471

**Published:** 2021-06-30

**Authors:** Jonny Engebø, Torbjørn Torsheim, Ståle Pallesen

**Affiliations:** ^1^Department of Psychosocial Science, University of Bergen, Bergen, Norway; ^2^The Norwegian Gaming Authority, Førde, Norway; ^3^Norwegian Competence Centre for Gambling and Gaming Research, University of Bergen, Bergen, Norway; ^4^Optentia, The Vaal Triangle Campus of the North-West University, Vanderbijlpark, South Africa

**Keywords:** channelization of gambling, gambling problems, gambling reforms, gambling regulation, prevention of gambling problems, substitution

## Abstract

The purpose of gambling regulation can be to ensure revenue for the public, to prevent crime and gambling problems. One regulatory measure involves restriction of what games can be offered in a market. In this study, the effects of two regulatory market changes are investigated: First, a restriction of availability when slot machines were banned from the Norwegian market in 2007, and second the introduction of regulated online interactive games to the same market in 2014. Data collected from the general population in the period from 2005 through 2018, comprising 2,000 respondents every year, are used to investigate how participation in gambling changed over time. The respondents were asked if they took part in various games or lotteries. Logistic regression analyses were used to predict the proportion participating in five groups of games and if changes in participation coincided with major market changes. The first change was associated with a reduction in gambling on slot machines as well as a reduction in gambling participation overall. Following the slot machine ban, results show an increase in women participating in games offered in land-based bingo premises. A general increase in gambling on foreign websites was also seen, albeit much smaller than the reduction in slot machine gambling. The increases can partly be explained as substitution of one type of gambling with another. New regulated online interactive games were introduced in 2014. Despite the relatively large growth of such games internationally, Norway included, increased online gambling in general and an increased marketing of foreign gambling websites, the participation on foreign websites seemed stable. However, the overall participation in online interactive games increased. The introduction of the regulated alternative seems to have had a channelizing effect. Overall, the changes in gambling participation coinciding with two major regulatory changes can be explained by transformations of physical and social availability, and in terms of mechanisms outlined by the model of total consumption.

## Introduction

Regulation of gambling serves several purposes. One is to ensure revenue for the public in terms of taxes or as income for good causes. Another is to prevent criminal actions related to gambling activities, whilst a third is to prevent gambling problems or to reduce negative consequences of gambling, for both individuals and societies (1).

Gambling accessibility can be regarded as physical, social and cognitive (2). Accordingly, gambling is prevented if a game is banned, hence no longer physically available, either land-based or online. If certain games are socially inacceptable, e.g., for family and friends, the threshold to participate is typically elevated (social accessibility). A high threshold for participation is also seen for games where gambling procedures or rules are difficult to comprehend (cognitive accessibility). In addition to accessibility, gambling is also affected by potential competing products. New gambling products may serve as substitutes for existing ones. Generally, “cannibalization” occurs when new products or services to a varying degree substitute existing products or services (3). From this it seems conceivable that removal of gambling products may lead to substitution by increased gambling on other gambling products.

Two major regulatory changes, relevant to accessibility and cannibalization, have taken place in Norway. The first concerned land-based gambling machines. In year 2001, the Norwegian gambling market had about 19,000 slot machines, operated by over 100 private operators with a wide distribution. The machines were available in open areas such as shopping centers, grocery stores and other public premises e.g., bus stations. At the time, public concerns about gambling addiction were growing. Coincidently, treatment providers reported an increase in people seeking help for gambling problems. As a response, the government decided to change the market for gambling machines to prevent gambling problems more efficiently, to enforce the age limit more strictly, and to prevent crime and fraud. Consequently, it was decided to allocate the state-owned operator, Norsk Tipping, a monopoly to operate gambling machines (4). This decision was however taken to court by the private operators. The case went through all three levels of the Norwegian court system and was also treated by the European EFTA court. Before the final verdict, note acceptors were banned from 1st of July 2006, hence only payment by coins was possible. The final verdict in the Norwegian supreme court ruled in favor of the government, and the removal of slot machines took place at the end of June 2007. The new machines, which were introduced mainly from 2009, were considered less harmful. These machines, interactive video terminals (IVTs), called Multix, were connected to a central server. A player card, and hence registered play, was required to gamble on these terminals. The mandatory player card enabled tools for prevention of problem gambling by enforcing an upper loss limit and other features where the gamblers could set further restrictions as well as self-exclude. In addition, the number of new gambling terminals was considerably lower and reduced to about one fifth of the former slot machine market (5).

A few studies investigated the effect of this regulatory change. Large school surveys among Norwegian teenagers aged 13–19 showed a significant decrease in participation at all levels (e.g., frequent gamblers, excessive gamblers) of gambling from before to after the ban of note acceptors (6). Indicators of problem gambling measured by SOGS-RA (7) and Lie/Bet (8) also showed a significant decrease from 2005 to 2006 (9). Changes in terms of gambling participation and indicators of gambling problems for teenagers has also been studied across a wider time interval. From 2002 to 2010 the overall gambling participation among teenagers was reduced from 78.5 to 64.3%. That study pointed to the slot machine reform as the main reason for the reduction. The same study also showed a reduction in problem gambling during the same period (10). In the adult population of former slot machine gamblers, 18 years and older, data for a panel study were collected twice in 2007, before and after the slot machine ban (*N* = 1,293). A significant reduction in overall gambling participation, gambling frequency and gambling problems was found (11). Another longitudinal study of adult participants collecting data before and after the ban, showed reduced gambling for half of the frequent slot machine gamblers, as they either reduced or completely ceased gambling (12).

Over the years, Norwegian gamblers have become engaged in interactive games on foreign web sites offered by operators without a Norwegian license. In order to channelize these gamblers to the Norwegian regulated market (13), Norsk Tipping introduced online interactive games in 2014. This represented the second regulatory change addressed in the present study. The online games offered encompass casino games (i.e., slot machines and table games except from poker), scratch games and bingo games. From the launch these games have been equipped with several responsible gambling (RG) tools, among others mandatory use of budget tools where gamblers must set personal loss limits. Also, an upper loss limit (maximal loss) is enforced (14). One panel study investigated the effect of the introduction of online interactive games by collecting data from a Norwegian sample (*N* = 5,809) aged 16–74 years in 2013, with follow-up data collection in 2015. Relatively few Norwegians gambled on online bingo or online casino in 2013. Of those who did, half did not gamble on such games in 2015. Half of those who still gambled on such games, gambled on the new regulated games. Hence, that study found some support for channelization from foreign websites to the new regulated internet-based casino games (15).

The total consumption model postulates an association between excessive or harmful consumption and total consumption in populations of gamblers. The model was originally derived from alcohol studies, but there are also studies showing that the model is valid for the gambling field (16). From this model one can expect that a reduction in the total amount of gambling in a market also will reduce the level of problem gambling (1). Thus, the model is relevant to understand the effects of regulatory changes toward gambling.

Previous research has addressed if the regulatory changes in the slot machine market, including the ban of note acceptors in 2006, led to changes in gambling behavior and gambling problems. However, there is a dearth of studies investigating the effect of introducing regulated interactive online games in 2014. Further, no study has been conducted covering the whole period of both changes, using the same type of data with samples from the entire population of gamblers. Consequently, the aim of this paper was to examine if and how gambling behavior changed after the two regulatory changes. This was done using data from regularly conducted surveys on gambling activity.

Specifically, the following research questions are addressed in the present study: (1) Can regulatory changes for specific games or game categories lead to changes in the participation of similar games? and (2) Can changes for some specific games lead to changes in the total consumption of gambling?

## Materials and Methods

Data used for this study were collected by surveying samples from the Norwegian population during 2005 through 2018. These data are used to predict if and to what extent participation in gambling activity has changed over the period. Data used to analyze gambling behavior were collected through semi-annual surveys, administered by an external research company on behalf of the Norwegian Gaming Authority. The surveys were based on phone interviews, landline and mobile. As the main goal of these surveys was to monitor developments in the gambling market, most survey questions have been unchanged during the whole period. This enables merging of all the raw data into one large datafile with about 28,000 respondents.

Except for new questions due to changes in the gambling market, there have not been any changes concerning the format, survey description, inclusion/exclusion criteria of participants or other aspects of the surveys. Due to procurement rules, a total of three analysis agencies have conducted the surveys, without changing the method, questions, or procedures. However, because mobile phones gradually have taken over for land-line phones, the proportion of mobile phone respondents has increased over the years. In the first survey (June 2005) used for this study, mobile phone users amounted to 29.1%. In the last survey (December 2018), 94.3% answered with mobile phones. The samples for land-line phone numbers were randomly selected from a database of land-line phone customers. The samples for mobile phone numbers were randomly selected from series of mobile phone numbers kept by The Norwegian Communication Authority.

Over the years response rates in surveys in general has decreased, also for phone-based surveys. This can be illustrated with an American example where *the contact rate* in a typical survey from Pew Research Centre decreased from 90% in 1997 to 62% in 2012, with a decline also in *the cooperation rate* (contacted persons who agreed to participate), from 43 to 14% over the same period. From this, *the overall response rate* is reduced from 36% in 1997 to 9% in 2012 (17). For most of the years, the exact response rates for the survey data in the present study are not reported, but the contact rates for the survey in June 2010 and an identical survey in June 2020 were accordingly 56% and 32%, whereas the cooperation rates were 19 and 13%, respectively. Hence the *overall response* rate was 10 and 4% when the persons never reached are taken into consideration. The survey in 2020 has a similar cooperation rate to the typical rate mentioned above in 2012. The overall response rate is however lower. The data used in the present study are weighed for age, gender, and place of residence (county) to match the demography of the adult Norwegian population. The data are used to explore predicted changes in gambling over time.

### Participants

Twice yearly, in June and December, a random sample of 1,000 Norwegians, aged 15 years or older, answered questions on which money games they have taken part in the last 12 months. See Table 1 for details about sample characteristics.

**Table 1:** Descriptive statistics of study variables (*N* = 28,251).

**Variable**	**Percentage**	***n***
Epochs		
1st (2005–2007)	22.1	6,243
2nd (2008–2013)	42.5	12,008
3rd (2014–2018)	35.4	10,000
Gender		
Female	50.3	14,217
Male	49.7	14,034
Age		
15–17 yrs.	4.0	1,140
18–24 yrs.	11.2	3,164
25–39 yrs.	24.7	6,984
40–59 yrs.	33.4	9,444
60 yrs. and older	26.6	7,519
Gambled on one or more game types		
No	23.7	6,700
Yes	76.3	21,551
Gambled on slot machines (to 2007) / IVT Multix (from 2009)		
No	94.7	26,742
Yes	5.3	1,509
Gambled in land-based bingo premises		
No	98.0	27,689
Yes	2.0	562
Gambled on foreign web sites		
No	95.8	27,066
Yes	4.2	1,185
Gambled on online interactive games, not poker		
No	97.2	27,455
Yes	2.8	796

*Data from every year from 2005 through 2018. n = 2,000–2,168 for each year. Mean/standard deviation (SD) for age: 45.94/(18.36)*.

### Procedure

In the study, gamblers have been categorized into different groups in order to address the research questions. One variable is general (gambled, on one or more game types vs. not gambled at all). In addition, four other specific gambling categories/variables are applied: (1) if gambled or not on land-based slot machines or IVTs, (2) if gambled or not on games in land-based bingo premises, (3) if gambled or not on foreign websites and finally, (4) if gambled or not on interactive online games (excluding online poker). The four specific categories have games with similar characteristics or include games such as the ones which were banned in 2007 (slot machines) or introduced in 2014 (online interactive games).

Except for trial licenses granted to two small operators which ceased in 2005, the foreign operators were alone to offer the latter type of games until 2014, when online interactive games were introduced to the regulated market by the Norwegian monopolist. This last variable reflects participation in online interactive games both before and after 2014. With logistic regression analysis, the data were used to predict participation in different games or game types.

### Instruments

#### Gambling Participation

The respondents were asked if they had gambled or not for each available money game or lottery in the Norwegian market, inclusive games offered by foreign operators. If the respondents endorsed minimum one game or lottery, e.g., betting, casino games or number games, they were categorized as gamblers under the general variable, gambled or not. Because of their relatively low “gambling factor,” two types of lotteries, small raffles without money prizes and a bottle recycling lottery, were not included in the variable. Gambling onboard ships in traffic between Norwegian and foreign harbors was first included in the survey from 2011 and is here included in in the general gambling variable. Number games are the most popular form for gambling in Norway, having the highest participation rate. As an example, and according to a national prevalence study in 2015, 77% of gamblers had participated in number games e.g., Lotto at least once during the last 12 months (18).

For the more specific variables the first was constructed for gambling or not on land-based slot machines or IVTs outside bingo premises. To be allocated to the first category, the gamblers had to confirm gambling on land-based slot machines, which were banned in 2007, the last 12 months in the survey period from 2005 through 2007 or on IVTs, from when these were introduced in 2009. The second specific variable is gambling or not on available games in land-based bingo premises where the respondents in order to be allocated to the first category had to confirm participation in at least one such game (i.e., traditional bingo, bingo machines, side games and slot machines or IVTs located in bingo premises). The third specific variable is gambling or not on games offered online by foreign operators. Games offered online by foreign operators comprise mostly of casino games, poker and sport betting, but also bingo, scratch games and horse racing. From the survey, gamblers who confirmed gambling online with other companies than Norwegian companies were allocated to the first category on this variable. The fourth and last specific variable concerned gambling or not on online interactive games regardless of whether this was offered by Norwegian or foreign operators. To be categorized as a gambler under this variable, the respondents had to confirm participating in online bingo, online slot machines or online table games (not poker), online scratch games or similar.

### Demographic

For this study we use data for gender and age. The total weighed sample was 50.3% female and 49.7 male (*N* = 28,251). Divided by age, 4.0% under 18 years, 11.2% in the age group 18–24 years, 24.7% in the group 25–39 years, 33.4% in the group 40–59 years and 26.6% 60 years or older (*N* = 28,251).

### Time

There are two time-variables. The first concerns year and continues from year 0 (2005) through year 13 (2018) where each level comprises 1 year. The second time variable is categorical and reflects three epochs, Epoch 1 (from 2005 through 2007), Epoch 2 (from 2008 through 2013) and Epoch 3 (from 2014 through 2018). In the analyses, the second (Epoch 2) is set as the contrast to both Epoch 1 and 3.

### Statistics

**Figure 1:**
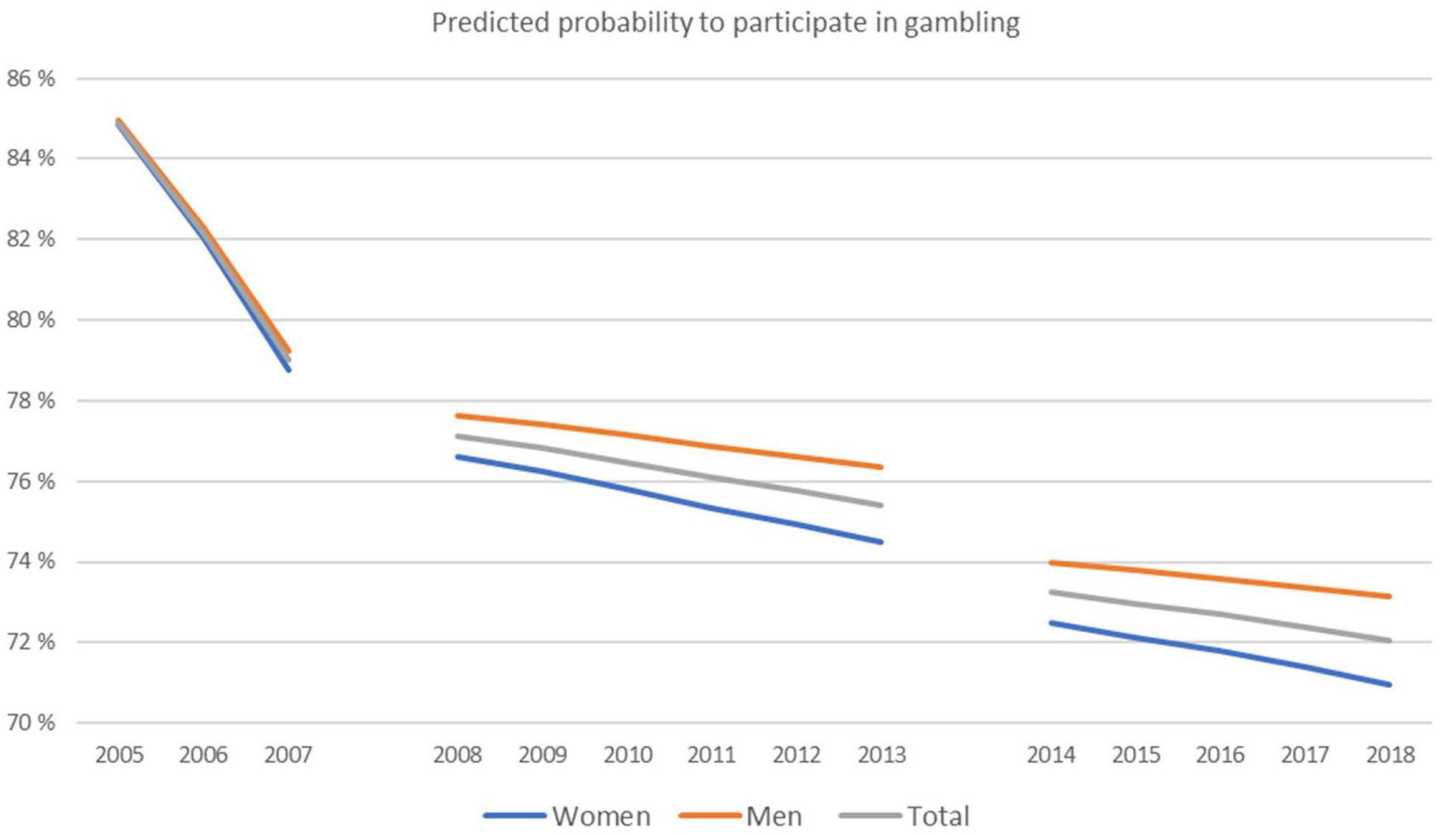
Predicted probability to participate in gambling, one or more games, overall and by gender (*N* = 28,251).

**Figure 2:**
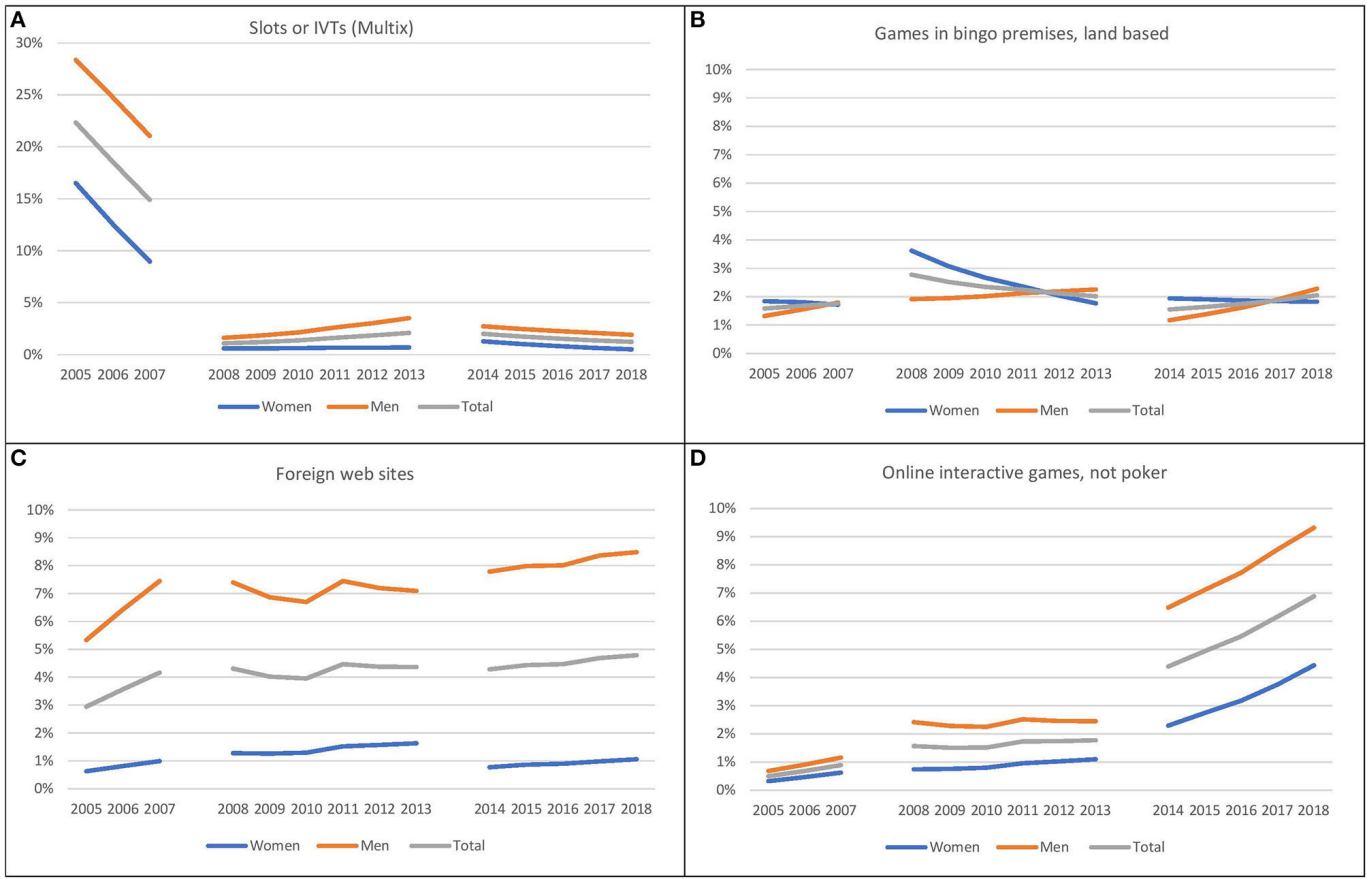
**(A–D)** Predicted participation in specific groups of games (*N* = 28,251). Panel **(A)** shows predicted participation on slot machines and IVTs (Multix), total and by gender, in three epochs (Epoch 1 from 2005 through 2007, Epoch 2 from 2008 through 2013 and Epoch 3 from 2014 through 2018). Panel **(B)** shows predicted participation on games in bingo premises, land-based, total and by gender, in three epochs (Epoch 1 from 2005 through 2007, Epoch 2 from 2008 through 2013 and Epoch 3 from 2014 through 2018). Panel **(C)** shows predicted participation on foreign websites, total and by gender, in three epochs (Epoch 1 from 2005 through 2007, Epoch 2 from 2008 through 2013 and Epoch 3 from 2014 through 2018). Panel **(D)** shows predicted participation in online interactive games, not poker, total and by gender, in three epochs (Epoch 1 from 2005 through 2007, Epoch 2 from 2008 through 2013 and Epoch 3 from 2014 through 2018).

In the statistical analyses, five different dichotomized (no = 0, yes = 1) dependent variables, each reflecting participation in the following gambling activities were included: Gambled or not on: (1) one or more available games, (2) land-based slot machines or ITVs Multix, (3) games in land-based bingo premises, (4) games offered from foreign web sites and, (5) online interactive games, but not poker. The descriptive statistics of the study variables are presented in terms of frequencies or mean and standard deviation. The data were further analyzed with logistic regression analyses adjusted for different variables. Independent variables comprised year (2005 = 0, 2006 = 1, …. 2018 = 13), epoch (2005–2007, 2008–2013 and 2014–2018, where 2008–2013 comprised the reference), gender (female = 0, male = 1) and age. In the first block, all independent variables were entered simultaneously, however without interaction terms. In the second block the interactions between year and epoch, and in the third block also the interaction between year and age, year and gender, epoch and age, and epoch and gender were added. Nagelkerke R-square was used to assess the explained variation of the different regression models. We have investigated potential multicollinearity between Time (year) and Epoch 1, 2, 3. All variance inflation factors (VIF) came out below 10, which is regarded as a threshold for problematic collinearity (19). The logistic regression analyses' predictions in gambling participation are presented in Table 2 and Figures 1, 2.

**Table 2:** Predicted probabilities (0–1), mean and standard deviation (*SD*) of participation in gambling per time epoch (*N* = 28,251, *n* = 6,243–12,008).

	**Epoch**	**Gambled (one or more of all games)**	**Slots and IVTs Multix**	**Bingo premises (land based)**	**Foreign web sites**	**Online interactive, not poker**
Mean	1 (2005**–**2007)	0.821	0.187	0.017	0.036	0.007
	2 (2008–2013)	0.763	0.015	0.023	0.042	0.016
	3 (2014–2018)	0.727	0.016	0.018	0.045	0.056
	Total	0.763	0.053	0.020	0.042	0.028
*SD*	1 (2005–2007)	0.025	0.140	0.007	0.046	0.006
	2 (2008–2013)	0.012	0.013	0.008	0.054	0.017
	3 (2014–2018)	0.018	0.012	0.006	0.061	0.045
	Total	0.039	0.097	0.008	0.055	0.036

## Results

Tables 1, 2 show that many have gambled at least once during the last year. Data which are not presented in these tables show that for the specific groups, a large majority of gamblers also participate within other games or game groups, varying from 92.5% for slot machines (participating in other games) and IVTs Multix (*n* = 1,509) to 98.7% (*n* = 796) for the online interactive games.

For two of the five variables examined, a reduction in participation from 2005 through 2018 was found: For gambling in total, the mean predicted probability was 82.1% for the first epoch (2005–2007), 76.3% for the second epoch (2008–2013) and 72.7% in the third epoch (2014–2018), respectively. The clearest reduction is for land-based slot machines and IVTs Multix. Here the mean predicted participation was 18.7% in the first epoch, and 1.5% and 1.6% in the two later epochs.

Gambling on foreign web sites and gambling on interactive games increased predicted participation and were for the three epochs, 3.6, 4.2, 4.5% and 0.7, 1.6, and 5.6%, respectively. The increase was strongest for online interactive games. The variable gambling in land-based bingo premises shows the lowest overall participation with predicted mean for the three epochs was 1.7, 2.3, and 1.8% respectively.

Logistic regression analysis was used to determine which effects the variables for time, gender and age had on the participation in gambling and changes between epochs. The analysis for each gambling variable was run in three blocks: In the first block, the impact of epoch, year, age, and gender was analyzed without including any interaction. In the second block, interactions between epochs and year were added. In the third block, interactions between both epochs and year by age and gender were added.

For two of the variables, gambling in total and gambling in land-based bingo premises, the analysis explained only 1.3 and 1.7% of the variation, respectively. For the other three variables, gambling on slot machines and IVTs Multix, foreign web sites and online interactive games the models explained far more of the variance, 31.5, 19.8, and 15.5%, respectively. Most of the explained variance was found in the first block.

**Table 4** shows that age and gender have a significant effect on all five variables after the first block. For gambling in total, participation increased with age, but for gambling on the four other specific gambling categories, participation decreased with age. For all game types but one, participation was higher for men. For bingo in land-based premises, participation was higher for women. The increased participation in overall gambling by age, most likely reflects a higher prevalence of older gamblers participating in number games. A prevalence study from 2015 showed that among the total amount of gamblers, 77% had participated in number games e.g., Lotto at least once during the last 12 months, and that participation rate for these games increased strongly with age (18).

After the first block, gambling in total participation was reduced from Epoch 1 to Epoch 2 (contrast). It was also reduced by year. For slot machines or IVTs Multix a significant decrease in predicted participation from Epoch 1 to Epoch 2 was found, whereas an increase was found from Epoch 2 to Epoch 3. Also, a reduction by year was found. For games in land-based bingo premises, an increase in predicted participation from Epoch 1 to Epoch 2 was found. The predicted participation on foreign websites was neither affected by year nor epoch. For online interactive games, an increased predicted participation per year was found and a lower participation in Epoch 1 compared to Epoch 2 as well as a higher participation rate in Epoch 3 compared to Epoch 2, and for the variable, the above-mentioned associations turned out non-significant when interactions were included. However, Table 3 shows that most of the variation is explained by the model without interactions.

**Table 3:** Accumulated explained variation (Nagelkerke R Square) and significance per block.

	**Gambled (one or more of all games)**	**Slots or IVTs Multix**	**Bingo in bingo premises, land-based**	**Foreign web sites**	**Online interactive, not poker**
	Nagelkerke/*p*	Nagelkerke/*p*	Nagelkerke/*p*	Nagelkerke/*p*	Nagelkerke/*p*
Block 1	0.011/0.000	0.307/0.000	0.013/0.000	0.196/0.000	0.153/0.000
Block 2	0.012/0.000	0.310/0.000	0.014/0.075	0.196/0.056	0.154/0.086
Block 3	0.013/0.131	0.315/0.000	0.017/0.041	0.198/0.013	0.155/0.101
Total	0.013/0.000	0.315/0.000	0.017/0.000	0.198/0.000	0.155/0.000

As Figure 1 shows, the overall predicted gambling participation was reduced over the years and there was a significant drop from Epoch 1 to Epoch 2. The epochs by year interaction shows that the reduction by year was strongest in Epoch 1.

For gambling on land-based slot machines / IVTs Multix there was also a reduction in participation. This is shown with a steep drop from Epoch 1 to Epoch 2. There was in addition a minor increase from Epoch 2 to Epoch 3. The epoch by year interaction shows there were reductions in participation by year in both Epoch 1 and Epoch 3, but not in Epoch 2. Younger people gambled more often on slot machines and IVTs Multix. As illustrated in Figure 2, the year by gender interaction showed a steeper reduction by year for males than females. The epoch by gender interaction is indicative of a similar decrease for both genders in Epoch 1, but that the predicted probability to gamble increases for male and not for female gamblers in Epoch 2.

For games in land-based bingo premises, there was a significant overall decrease by year and a significant lower participation in the first epoch compared to the second. Further, and as illustrated in Figure 2 and Table 4, the model predicted higher probability to gamble amongst women and younger subjects, compared to men and older ones. The epoch by year interaction shows the participation decreased by year in the second epoch but increased in the third epoch. Gender also interacted significantly with year and the epochs. The share of male gamblers increased, and the share of female gamblers decreased by year. The epoch by gender interaction showed a steeper increase for women than males from Epoch 1 to 2 whereas the opposite development was found from Epoch 2 to Epoch 3.

**Table 4:** Logistic regression analyses of five gambling variables in the Norwegian gambling market year 2005–2018.

		**Gambled (one or more of all games)**		**Slots and IVTs Multix**		**Bingo in bingo premises**		**Foreign web sites**		**Online interactive, not poker**	
		***B***	***S.E*.**	***B***	***S.E*.**	***B***	***S.E*.**	***B***	***S.E*.**	***B***	***S.E*.**
Block 1	Year	−0.028	0.010^**^	−0.084	0.027^**^	0.020	0.029	0.022	0.021	0.092	0.025^***^
	Epoch 1	0.229	0.059^***^	2.380	0.142^***^	−0.459	0.173^**^	−0.182	0.129	−0.522	0.208^*^
	Epoch 3	−0.037	0.061	0.461	0.184^*^	−0.173	0.185	−0.089	0.134	0.772	0.160^***^
	Age	0.002	0.001^*^	−0.044	0.002^***^	−0.017	0.002^***^	−0.059	0.002^***^	−0.044	0.002^***^
	Gender (f = 0. m = 1)	0.076	0.028^**^	0.911	0.061^***^	−0.194	0.086^*^	1.960	0.087^***^	0.910	0.080^***^
Block 3	Year	−0.013	0.029	−0.072	0.090	−0.218	0.082^**^	−0.024	0.073	0.067	0.079
	Epoch 1	0.723	0.183^***^	3.933	0.532^***^	−1.171	0.497^*^	−1.324	0.462^**^	−1,119	0.659
	Epoch 3	−0.251	0.250	3.378	0.808^***^	−0.595	0.788	−0.901	0.635	−0.761	0.656
	Age	0.003	0.003	−0.041	0.011^***^	−0.024	0.010^*^	−0.074	0.010^***^	−0.057	0.011^***^
	Gender (f = 0. m = 1)	0.038	0.114	0.532	0.386	−1.240	0.336^***^	1.964	0.350^***^	1,364	0.354^***^
	Epoch 1 by Year	−0.180	0.042^***^	−0.359	0.062^***^	0.139	0.126	0.218	0.091^*^	0.286	0.195
	Epoch3 by Year	0.004	0.020	−0.246	0.073^**^	0.135	0.065^*^	0.037	0.045	0.101	0.053
	Year by Age	0.000	0.001	0.002	0.002	0.002	0.002	0.002	0.002	0.000	0.002
	Year by Gender	0.008	0.019	0.138	0.060^*^	0.185	0.058^**^	−0.053	0.058	−0.071	0.055
	Epoch 1 by Age	−0.006	0.003	−0.012	0.010	−0.001	0.010	0.019	0.009^*^	0.012	0.014
	Epoch3 by Age	0.003	0.003	−0.015	0.010	−0.009	0.011	−0.007	0.010	0.016	0.011
	Epoch 1 by Gender	−0.029	0.116	0.202	0.348	0.875	0.345^*^	0.212	0.359	−0.654	0.438
	Epoch 3 by Gender	−0.031	0.122	−1.035	0.393^**^	−0.952	0.390^*^	0.914	0.378^*^	0.351	0.361

*Results for the first and third block, before and after interactions between variables were included. Epoch 2 is the contrast to Epoch 1 and 3. (N = 28,251)*.

* *p < 0.05*,

** *p < 0.01*,

*** *p < 0.001*.

The participation on foreign websites increased slightly over the years. The strongest increase was seen in Epoch 1. In Epoch 2, yearly change was not statistically significant. However, there was a significant lower participation in Epoch 1 compared to Epoch 2. Younger persons and males had a higher predicted probability to gamble on such websites. The epochs by year interaction showed that the increase by year was steeper in Epoch 1 than Epoch 2. The epoch by age interaction reflects that the effect of age on participation increased from Epoch 1 to Epoch 2. The epoch by gender interaction suggested a slight reduction in gambling participation from Epoch 2 to Epoch 3 for females, whereas an increase was found for males.

Also, for gambling on interactive online games an increase in participation was detected from Epoch 1 to Epoch 2. Men and young subjects had a higher predicted probability to gamble on online interactive games than women and older subjects. Table 4 (block 1) show a significant increase from Epoch 2 to Epoch 3 on interactive online games.

## Discussion

In general, the total predicted participation in gambling decreased over the years. Also, from Epoch 1 to Epoch 2 a significant reduction was found. The same was true for slot machine gambling, although here an increase from Epoch 2 to Epoch 3 was found. The introduction of new interactive games in 2014 did not lead to an increase in the overall gambling participation as most of these gamblers already participated in other games.

The reduction in overall gambling participation reported here coincides with changes in two of the other Nordic countries. Survey data from The Public Health Agency of Sweden show a reduction in gambling participation from 2005 to 2018. However, the reduction in Sweden^1^ seems to be more gradual than the Norwegian which clearly was steepest from 2005 through 2007. In Denmark, a reduction in gambling participation from 2005 to 2016 is reported (20). Data from the two other Nordic countries show increases in the similar period. In Finland, an increase from 2007 to 2015 followed by stabilization from 2015 to 2019 has been reported (21). In Iceland, which experienced an economic crisis in 2008, an increase from 2005 and 2007 to 2011 was found (22). New Zealand is an example of another country with decreased general gambling participation in the same period (23).

For the general participation, the Norwegian reduction is steeper in the first epoch than in the second. The first epoch covers the regulatory changes for the slot machines. Since the gamblers who played on slot machines very often also gambled on other games, the reduction in overall gambling must have other explanations than that people no longer gambled on slot machines. One reason can be that the reduction is a general trend which is also seen in other countries without the same regulatory changes. Another explanation is that some of the slot machine gamblers stopped gambling altogether due to the slot machine reform.

In 2005, the revenue (GGR) from slot machines accounted for 45% of the total in the Norwegian market^2^. The current regression analysis predicted that between 20 and 25% of the population had gambled at least once on slot machines in 2005. The overall gambling prevalence the same year was 85% and a large majority of gamblers on slot machines also played other games, thus illustrating that the slot machines' contribution to the total revenue was high compared to other games.

The total consumption theory emphasizes an association between excessive or harmful consumption and the total consumption in a population (16). The revenue figures for slot machines show that this was a game with relatively high revenue, and as such a game type with more excessive gambling. From the total consumption theory one can expect that a reduction in the total amount of gambling in a market also will reduce the level of problem gambling (1). Figures from the national helpline for problem gamblers can serve as indicators for how gambling problems develop. In the period of regulatory changes for slot machines the reduction in calls was nearly 70%, from 2,100 in 2005 to 657 in 2008^2^ which is a relatively larger drop than the reduction in the total revenue (GGR) of 33% (from NOK 11.7 billion in 2005 to NOK 7.9 billion in 2008).^2^ The reduction in problem gambling at that time was also seen in results from prevalence studies. A prevalence study conducted in 2013 compared the results with previous Norwegian studies using the same instrument (Problem Gambling Severity Index; PGSI) (24), and suggested a reduction in gambling problems, especially related to the low-risk gambler and the problem gambler categories (25). The prevalence rates for moderate risk and problem gamblers (PGSI 3+) were as follows: 5.5% (2005), 4.3% (2007), 3.6% (2008), 4.4 % (2010), and 3.0% (2013). It should be mentioned that the reported prevalence rates (PGSI 3+) for the two subsequently studies were 3.2% (2015) and 4.5% (2019), respectively, (26). Notably, the survey populations and the data collection procedures were not identical for all these studies (2005–2019), thus comparisons should be done with caution.

The findings support the total consumption model (1) in two ways. The first concerns the finding that the slot machine ban also led to a decrease in other forms of gambling. This was also seen in the panel study which found a significant reduction in the overall gambling participation for slot machine gamblers after the ban in 2007 (11). The steep drop in gamblers seeking help from the national helpline for problem gamblers compared to the reduction of the gambling revenue supports at least indirectly the association between excessive or harmful gambling and total gambling consumption. A similar association was shown in UK when the average gambling expenditures were doubled with the introduction of a national lottery, and the proportion of households where gambling expenditure was excessive increased four-fold (27).

When studying specific games or groups of games, the development of participation varies from the participation in general. At the same time as the regulatory restrictions on slot machines were introduced, the analysis shows a dramatical drop in the prevalence of slot machine gamblers through the first epoch. The reduction coincides firstly with the ban on note acceptors in 2006 and then the ban on the slot machines in 2007. For games in land-based bingo premises, which initially had a very low participation rate, the results predicted a significant increase in the female participation from the first to the second epoch.

Online gambling indicates an increase in the participation on foreign websites from Epoch 1 to Epoch 2 when all interactions were added to the model. The increase by year in Epoch 1 is significantly higher than the change by year in Epoch 2. Also, at the general level the participation in gambling on foreign websites was higher in Epoch 2 than Epoch 1. For gambling on interactive online games, the participation was lower in Epoch 1 than in Epoch 2. There was also predicted a significant increase from the second to the third epoch for the interactive games when the interaction terms were not taken into consideration.

Changes in participation which coincide with regulatory changes can be explained with change in accessibility. With the ban on slot machines, this form of gambling became inaccessible and hence a large drop in participation was predicted. The lower participation rate for the gambling machines (IVTs Multix) which replaced the former slot machines results from lower accessibility, e.g., fewer machines, stricter regulation for placement and the requirement of player identification. A suggested explanation to the increase in participation for games in bingo premises is that games with similar characteristics as the old slot machines remained in such premises. The predicted increase is however smaller than the reduction for slot machines. For online gambling, the introduction of regulated online interactive games and market trends are likely plausible explanations for the increase from Epoch 2 to 3. The increase is significant when the interaction terms were not added to the model, then also year was positively associated with participation. Still, it should not completely be ruled out that stricter regulation for the land-based slot machines through Epoch 1, partly led to more participation on online interactive games. For gambling on foreign web sites, the analysis further predicted a steeper increase by year in Epoch 1 than in Epoch 2. Similar to games in land-based bingo premises, the increase for online gambling is much weaker compared to the reduction for slot machines.

Only foreign operators offered online games such as slot machines or other interactive games through Epoch 2. However, in the beginning of Epoch 3 (2014), Norwegian interactive online games were introduced as a regulated alternative. From the analysis, there is no predicted reduction in the overall participation with the foreign operators that coincides with the introduction of national regulated online interactive games. However, the introduction of the regulated interactive games, together with regulatory measures aimed toward foreign operators, may have prevented a growth in the participation on foreign operators' websites. Two gambling market consultant companies estimated a large growth for the online interactive games, casino and bingo, poker not included. The estimated increase in revenue for Europe from 2013 to 2018 was 65 and 93%^3^^,^^4^. Nationally, three subsequent prevalence studies in Norway, with data from 2013, 2015, and 2019 show an increase in online gambling. Subsumed across all games, the prevalence of online gambling, at least once during the last year, has increased by each study, involving 25.8% (2013), 29.2% (2015) and 58.3% (2019) of the gamblers, respectively (18, 26). The strongest increase took place between 2015 and 2019. This was especially pronounced for mobile phones, by which online gambling increased from 17.0 to 48.7% (26). Further, the foreign operators significantly increased their spending on TV-marketing aimed for the Norwegian population in the third epoch^5^. Despite these trends and market efforts, an increase was not found in the present study's prediction of gambling participation on foreign websites. Coincidentally regulatory measures restricting the foreign operators most likely also prevented growth of gambling on the foreign websites (e.g., banning of marketing and payment services). These restrictive measures have been further developed after 2018. In 2019, the ban on payment services became more efficient and from 2021, the ban on marketing will also include the intermediaries of advertising broadcasted from abroad. DNS warning/blocking has not yet been implemented^2^.

The increased participation rate for interactive online games from Epoch 2 to 3 can thus have at least two explanations: One is the general and international trend where such games seem to have increased in popularity and the shift to more gambling with electronic devices, especially mobile phones. The second explanation concerns increased physical and social availability of a regulated alternative to the foreign websites.

Looking at the participation on foreign websites, there is a relatively small, but significant change for gender where the female shares of gamblers on such sites decreased significantly from the second to the third epoch. A suggested interpretation for this change is that women, to a larger degree than men, have moved their gambling from foreign websites to the regulated Norwegian website. The regulated games introduced in 2014, were launched with several measures to prevent excessive gambling and reduce harm (e.g., stricter limits for stakes and maximum loss limits). This interpretation is in line with research showing that women are more positive to measures which prevent gambling problems and reduce negative consequences (28, 29). Another study has shown that women take less risks than men and judge the negative consequences of gambling as more likely to occur and as more severe (30). For some gamblers, national regulation of games with stricter measures to prevent problem gambling can thus appear more socially acceptable than the foreign operators' websites.

With their characteristics, the interactive online games (i.e., scratch games, bingo or casino games) have relatively higher risk for problem gambling. Among the characteristics recognized to increase the risk of problems are event frequency and availability^6^ (31). Several of the characteristics are relevant for interactive online games. As previous mentioned, national prevalence data concerning problematic gambling (PGSI 3+) showed an increase from 2015 to 2019 (from 3.2 to 4.5%) (26). The authors mention increased participation in games with higher risk and increased use of mobile phones as gambling device as two of the possible causes for the increase in problematic gambling (26). The helpline had in 2019 a 12% higher rate of contacts compared to 2015^2^ (764 and 680, respectively).

### Practical Implications

The present study shows that regulatory measures which change accessibility to gambling opportunities impact gambling participation. Such changes may have a direct effect on problem gambling related to specific games, and indirectly through the mechanisms predicted by the total consumption model, where a reduction in overall gambling will reduce the prevalence of problem gambling.

Social accessibility should also be acknowledged as a relevant term in the regulation of gambling as this can affect participation. In line with international trends, and increased use of electronic devices for gambling, the participation in online interactive games has increased in Norway. This is however not visible for participation on foreign websites. Moreover, a reduction in the share of female gamblers on foreign website from Epoch 2 to Epoch 3 was found. One explanation can be that some of these prefer a regulated alternative equipped with several measures to reduce risk or harm. Another example which can illustrate social acceptance and hence accessibility is the reduction in gambling participation from 2005 through 2007 on slot machines. One explanation for a drop in 2006 is the ban on note acceptors which restricted the payment method to coins. Another explanation could be that the focus on gambling problems in society in general, which led to the ban on slot machines, also led to a reduced motivation or interest to gamble.

In addition to implications regarding availability, the mechanisms of the total consumption model should also be recognized where regulatory changes for one type of game could lead to changes for other games. Changes can also affect the level of extensive gambling or gambling problems.

### Strengths and Limitations

To our knowledge, no previous study has used trend data for gambling covering all types of gambling participation within a jurisdiction over 14 years (2005–2018). With this it has been possible to analyze two regulatory changes based on the same data set. Other assets are the regularity of the surveys (conducted in June and December) preventing season as a confounder. The data also covered a minimum of 3 years before and after each regulatory market change, which thus partly compensate for the lack of control conditions/groups and comprises as such a quasi-experimental interrupted time-series design (32). However, it would have been a strength to compare Norwegian data with temporally similar data from other countries that did not implement the same regulatory changes. Nevertheless, we refer to comparable data on general trends from other Nordic countries. It should also be mentioned that each survey comprised at least 1,000 respondents which is considerable sample size.

One limitation of the present study is that survey data collected over telephone have low response rates. However, studies have showed that the response rate on its own is not a good predictor of non-response bias or low validity (33–35). Low response rate can be a disadvantage as regards representativeness. However, if results systematically under- or overestimate the prevalence of gambling participation it is still possible to study trends if the reasons for non-response do not change over time.

The fact that our study is mainly based on self-report and cross-sectional data is a noteworthy limitation. Ideally, panel data should have been used where the same respondents participated over time. This would however be difficult to achieve for such a wide timeframe as in the present study. Such an approach would also exclude analysis of new and young gamblers and old gamblers would naturally fall out of such panel.

It can be difficult to isolate the effects of various regulatory measures on gambling behavior. Among others, questions may be raised as to whether the change in gambling behavior was caused by the ban on note acceptors in 2006 or the ban on slot machines in 2007. The publicity and discussions at the time about slot machines could also have affected gambling behavior. Both the note acceptor ban, the slot machine ban and the later launch of new gambling terminals were part of the reform of the slot machine market. Conclusions about causality are also limited for the new and regulated online interactive games launched in 2014. Due to the design of the present study, it is not possible to determine how much of the change in behavior is caused by the launch of the games, the restrictive regulatory measures, a change in how people prefer to gamble or factors such as social acceptability.

In our view, our study suggest causality between market events and behavioral changes, but we cannot rule out other explanations for our findings. The analysis with highest explained variation is 31.5%. The study investigated if there were any changes in gambling behavior that coincide significantly in time with the two major regulatory changes in 2007 and 2014.

## Conclusion

With the implementation of the ban on note acceptors in 2006 followed by the total ban on slot-machines in 2007 and the consequently reduction in gambling on these machines, a reduction in general gambling participation was also detected. Simultaneously, the data analysis predicted a certain increase in female participation in games in land-based bingo premises and a general increase in gambling on foreign websites following the ban. These increases were however much smaller than the reduction seen for slot machines. The actual increase in gambling in bingo premises and foreign web sites from Epoch 1 to 2 can still, at least partly, be explained as a substitution where some gamblers moved their gambling to arenas which offered similar games.

When new regulated interactive games were introduced in 2014, the overall participation in online interactive games increased, but the participation on foreign websites seemed stable. Despite the relatively large general growth for such games internationally, also in Norway, increased prevalence of online gambling and increased effort to market foreign gambling websites, the introduction of the regulated alternative seems to have had a channelizing effect. No increase in participation was visible for the foreign operators. This channelizing effect was relatively stronger for women than men.

Overall, the changes shown in our analysis coinciding with two major regulatory changes of the gambling marked in Norway can be explained by the transformations of physical and social availability, and in terms of mechanisms outlined by the model of total consumption.

## Data Availability Statement

Data are available on request. Requests to access the dataset should be directed to Jonny Engebø, jonny.engebo@lottstift.no.

## Ethics Statement

Ethical review and approval was not required for the study on human participants in accordance with the local legislation and institutional requirements. Written informed consent from the participants' legal guardian/next of kin was not required to participate in this study in accordance with the national legislation and the institutional requirements.

## Author Contributions

JE has conducted the analysis and drafted the first version of the manuscript. All authors contributed to the interpretation of data, revised the work critically for important intellectual content, approved the final version to be published, and agreed to be accountable for all aspects of the work in ensuring that questions related to the accuracy or integrity of any part of the work are appropriately investigated and resolved.

## Conflict of Interest

JE works as a senior adviser with The Norwegian Gaming Authority where one of his major tasks is related to regulation and responsible gambling. He is also a Ph.D. candidate with the University of Bergen. In addition, JE is a board member of GREF (Gaming Regulators European Forum) and he is also co-chair of a GREF working group in responsible gambling. Further he is a member of the executive committee of EASG (The European Association for the Study of Gambling). The remaining authors declare that the research was conducted in the absence of any commercial or financial relationships that could be construed as a potential conflict of interest.

## Footnotes

^1^The Public Health Agency of Sweden. Available online at: https://www.folkhalsomyndigheten.se/folkhalsorapportering-statistik/statistikdatabaser/folkhalsodata-och-folkhalsostudio/ (accessed February 15, 2021).

^2^The Norwegian Gaming Authority. Available online at: www.lottstift.no (accessed February 15, 2021).

^3^Global Betting & Gaming Consultants. Available online at: https://www.gbgc.com

^4^Gambling Capital. Available online at: https://h2gc.com

^5^The Norwegian Media Authority. Available online at: https://www.medietilsynet.no (accessed February 15, 2021).

^6^Gamgard. Available online at: www.gamgard.com (accessed February 15, 2021).
